# The inflammatory response to extracorporeal membrane oxygenation (ECMO): a review of the pathophysiology

**DOI:** 10.1186/s13054-016-1570-4

**Published:** 2016-11-28

**Authors:** Jonathan E. Millar, Jonathon P. Fanning, Charles I. McDonald, Daniel F. McAuley, John F. Fraser

**Affiliations:** 1 Critical Care Research Group, University of Queensland, Brisbane, Australia; 2 Wellcome-Wolfson Centre for Experimental Medicine, Queen’s University Belfast, Belfast, UK; 3 Critical Care Research Group, The Prince Charles Hospital, Rode Road, Chermside, Queensland 4032 Australia

**Keywords:** Extracorporeal membrane oxygenation, ECMO, Inflammation, SIRS, Coagulation

## Abstract

Extracorporeal membrane oxygenation (ECMO) is a technology capable of providing short-term mechanical support to the heart, lungs or both. Over the last decade, the number of centres offering ECMO has grown rapidly. At the same time, the indications for its use have also been broadened. In part, this trend has been supported by advances in circuit design and in cannulation techniques. Despite the widespread adoption of extracorporeal life support techniques, the use of ECMO remains associated with significant morbidity and mortality. A complication witnessed during ECMO is the inflammatory response to extracorporeal circulation. This reaction shares similarities with the systemic inflammatory response syndrome (SIRS) and has been well-documented in relation to cardiopulmonary bypass. The exposure of a patient’s blood to the non-endothelialised surface of the ECMO circuit results in the widespread activation of the innate immune system; if unchecked this may result in inflammation and organ injury. Here, we review the pathophysiology of the inflammatory response to ECMO, highlighting the complex interactions between arms of the innate immune response, the endothelium and coagulation. An understanding of the processes involved may guide the design of therapies and strategies aimed at ameliorating inflammation during ECMO. Likewise, an appreciation of the potentially deleterious inflammatory effects of ECMO may assist those weighing the risks and benefits of therapy.

## Background

Extracorporeal membrane oxygenation (ECMO) is a technology capable of providing short-term mechanical support to the heart, lungs or both. Despite having first been employed clinically in the 1970s [1], the more widespread use of ECMO in critically ill adult patients is a recent phenomenon [2]. Over the last decade, the number of centres offering ECMO has grown rapidly. At the same time, the indications for ECMO in adults have expanded beyond acute severe respiratory and cardiac failure [3] to include extracorporeal cardiopulmonary resuscitation (ECPR) [4] and as a bridge to lung transplantation [5]. Due to its origins, the use of ECMO is much better established in neonatal and paediatric populations [6]. In fact, neonatal ECMO for respiratory failure accounts for 43% of the Extracorporeal Life Support Organisation (ELSO) registry, comprising 25 years of records of paediatric and adult patients undergoing ECMO [7].

Survival rates in neonates and children supported with ECMO, especially in cases of respiratory failure, are generally high [8]. For adults, it is difficult to quantify the mortality of patients undergoing ECMO. Several studies have varied in their reported mortalities based on indication and modality [8, 9], ranging from 76% in one cohort undergoing ECMO and dialysis [10] to 37% in a mixed veno-venous (VV)/veno-arterial (VA) ECMO group [11]. The ELSO registry, between 1989 and 2014, reports mortalities for respiratory and cardiac ECMO, of 57% and 41% respectively [7]. In addition, there are a number of complications that may occur, any of which is capable of inflicting serious morbidity. These can be broadly separated into those related to the ECMO device (e.g., oxygenator or pump malfunction, circuit clotting, cannula issues) and physiological complications (e.g., bleeding, haemolysis, and infection). In this review, we examine the pathophysiology of one less well-recognised complication, the inflammatory response to ECMO.

## The inflammatory response to ECMO

**Fig. 1:**
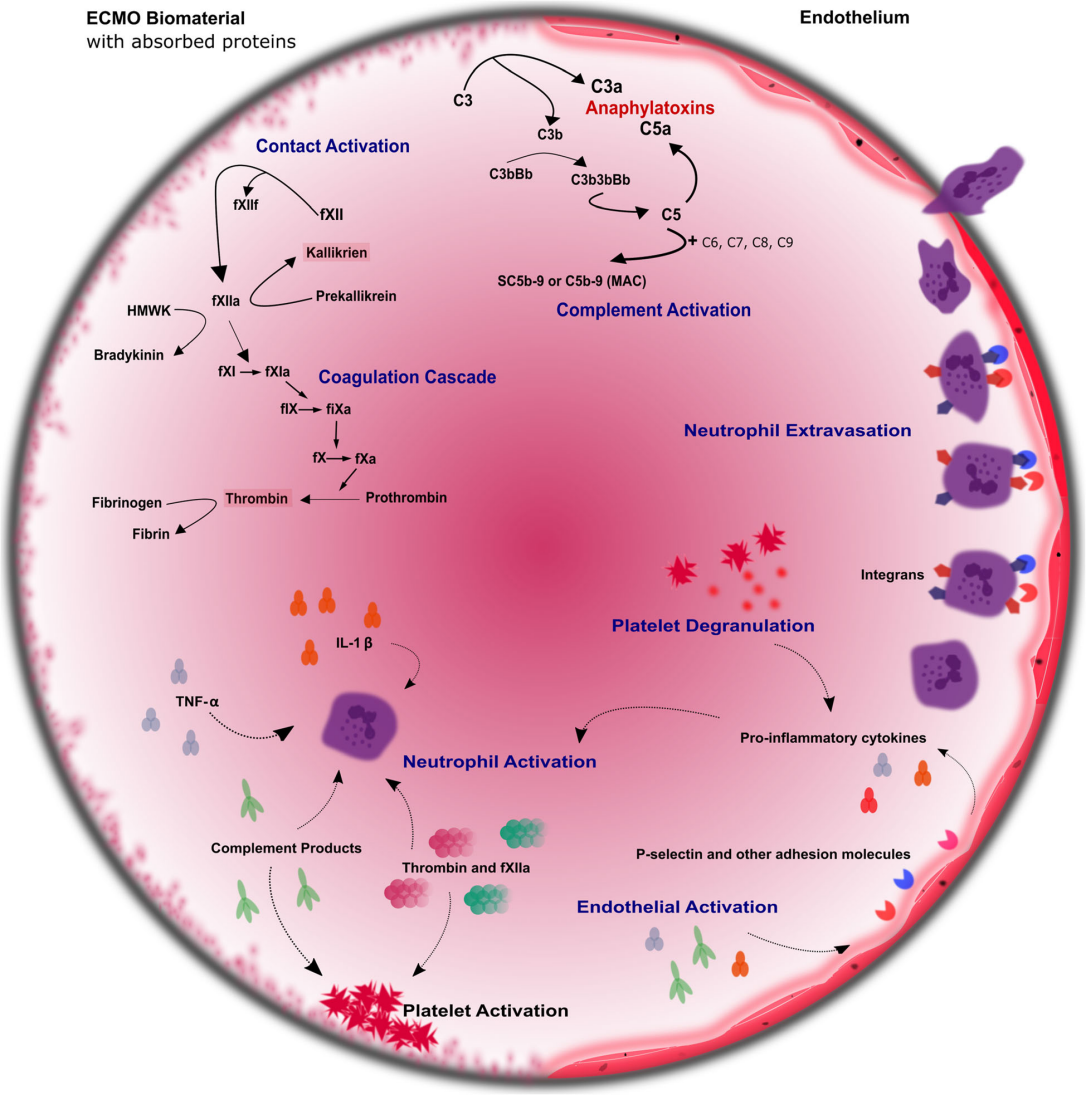
The inflammatory response to extracorporeal membrane oxygenation (*ECMO*). During ECMO, the complement and contact systems are activated as a result of blood-biomaterial interaction. The alternative complement pathway (AP) is primarily responsible for producing the anaphylatoxins C3a and C5a and the membrane attack complex (*MAC*). This occurs as the result of increased hydrolysis of C3 on the biomaterial surface. The contact system is responsible for producing activated factor XII (*FXIIa*), which induces the intrinsic coagulation pathway, leading to thrombin formation. Products produced by each of these systems, promote the production of pro-inflammatory cytokines and have direct effects on leukocytes, platelets and the vascular endothelium. In particular, neutrophils are activated, leading to increased neutrophilic infiltration of tissue and eventual organ damage

The initiation of ECMO is associated with an immediate and complex inflammatory reaction, similar to that seen in systemic inflammatory response syndrome (SIRS) [12]. At that moment when the patient’s blood first comes into contact with the foreign surface of the extracorporeal circuit, a variety of coagulative and inflammatory cascades are activated (Fig. 1). Levels of pro-inflammatory cytokines rise rapidly [13–19], which, in association with activation of the complement and contact systems [20–25], results in leukocyte activation [26–28]. This innate immune response, if severe, persistent or unchecked by a compensatory anti-inflammatory response (CARS) [29], may lead to endothelial injury, disrupted microcirculation, and end-organ dysfunction [30–35]. Despite major improvements in pump and circuit design, oxygenators and the advent of heparin-bonded surfaces, the SIRS response to ECMO remains a clinical concern. While a large volume of work has been directed towards elucidating and targeting the inflammatory response to cardiopulmonary bypass (CPB) [36–38], a closely related form of extracorporeal circulation, much less has been devoted to studying the inflammatory response to ECMO.

## Differences between ECMO and CPB

ECMO and CPB are closely related and share several similarities. Both techniques generate a biomaterial-induced inflammatory response in patients. Despite this, there are important distinctions between ECMO and CPB (Table 1), of which anyone attempting to infer from one to the other must be aware.

**Table 1:** Key differences between extracorporeal membrane oxygenation (ECMO) and cardiopulmonary bypass (CPB)

	ECMO	CPB
Duration	Days to weeks	Minutes to hours
Anticoagulation	Low-dose heparin	High-dose heparin
Reversal of anticoagulation	Not used	Protamine administration
Haemodilution	Yes - to a lesser degree than CPB	Yes
Hypothermia	No	Yes
Air-blood interface	No - closed circuit	Yes - some closed variants exist
Pulsatility	Variable, dependent on mode	Non-pulsatile

The most obvious distinction between ECMO and CPB is in the duration of support provided. CPB is commonly employed for only minutes to hours to facilitate a surgical procedure. Conversely, ECMO, used in patients with severe organ failure, can be prolonged for weeks to months. This difference in duration requires a different approach to anticoagulation. During CPB, loading doses of unfractionated heparin between 300 and 500 U/kg may be used [39], versus 40–80 U/kg during ECMO [40]. Upon the completion of CPB, protamine sulphate is administered to reverse the effects of heparin, a practice which is avoided during and at the conclusion of ECMO. This is an important distinction, given that the formation of protamine-heparin complexes is known to exacerbate the inflammatory response (via activation of the classical and lectin complement pathways) [41, 42].

Hypothermia is mandatory during CPB, but not commonly employed during ECMO. Likewise, haemodilution, which may also be employed during CPB, is not seen to the same degree during ECMO. Large observational studies have demonstrated an association between the lowest haematocrit recorded during CPB and post-operative mortality [43]. One suggested explanation for this is that haemodilution leads to increased neutrophil activation [44].

As part of the surgical nature of conventional CPB, cardiotomy suctioning, venting of blood and venous reservoirs are incorporated into circuits. This introduces a blood-air interface. Multiple studies have detected higher levels of pro-inflammatory cytokines in cardiotomy-suctioned blood and, in some instances, lower levels of anti-inflammatory cytokines, such as IL-10 [45–47]. The absence of an air/blood interface during ECMO may be a factor in reducing the inflammatory response.

Commonly, perfusion during CPB is non-pulsatile [48]. During VA-ECMO, depending on the residual function of the native heart, varying degrees of pulsatile flow are generated in the face of retrograde arterial perfusion by the ECMO system. This is not the case in VV-ECMO, which uses an ‘“in series configuration’, relying on the native heart for systemic perfusion [49]. Evidence from CPB suggests that pulsatile flow during extracorporeal circulation may act to reduce the inflammatory response [50]. It has been hypothesised that this may be due to the ability of pulsatile perfusion to better sustain the functional state of the microcirculation [51].

Contrary to ECMO, CPB involves inflicting an ischaemia-reperfusion injury [36]. Clamping of the aorta during surgery renders the heart, and to a large extent the lungs, ischaemic. At the completion of surgery, this clamp is removed and both organs undergo a period of reperfusion. Each phase causes a significant inflammatory reaction. In some patients this may prove significant, leading to the onset of lung ischaemia-reperfusion injury or so-called pump lung [52].

Finally, there are clear patient differences. The majority of patients undergoing CPB will have chronic disease (often severe), but are unlikely to be acutely unwell at the time of the procedure. Conversely, those undergoing ECMO are usually critically ill.

## The pathophysiology of inflammation during ECMO

The inflammatory response caused by ECMO occurs as a reaction to the exposure of blood to the extracorporeal circulation. Both systemic and cellular factors initiate and propagate the SIRS-like cascade. These include several humoral and cellular systems, most notably the contact, intrinsic and extrinsic coagulation, and the complement systems and endothelial cells, leukocytes, platelets and cytokines. Here we will consider each in turn, although it should be borne in mind that these systems are largely interdependent and still not fully understood.

## The interplay between coagulation and inflammation

### The contact system

The contact system is composed of several associated plasma proteins: factor XII (Hageman factor), factor XI, high molecular weight kininogen (HMWK) and prekallikrein (Fletcher factor). When blood, in which these proteins are circulating, comes into contact with the extracorporeal circulation, factor XII is cleaved into two proteases: factor XIIa and factor XIIf. Factor XIIa converts prekallikrein into active kallikrein and HMWK into bradykinin (Fig. 1). This is a rapid process, with Factor XIIa activity in the ECMO circuit reaching maximal levels within 10 minutes of initiation [53]. In addition to their role promoting coagulation, kallikrein and bradykinin drive inflammation. Kallikrein production during ECMO is known to directly activate neutrophils [54]. More recently, neutralisation of factor XIIa using a novel inhibitory antibody has been shown to reduce inflammation in ex-vivo and animal models of ECMO [55].

Bradykinin has pleiotropic effects, which include stimulating the release of nitric oxide, tumour necrosis factor-alpha (TNF-α) and IL-10 [56]. Little is known about bradykinin activity during ECMO, although CPB is associated with dramatically elevated levels [57]. A major contributory factor in this is that the lungs, bypassed during CPB, are the major site of bradykinin inactivation. This is not likely to be a comparable issue during VV-ECMO, but may play a role in a VA-ECMO configuration.

### Intrinsic and extrinsic coagulation

Contact system activation eventually leads to triggering of the intrinsic coagulation pathway. Factor XIIa, formed as a product of contact activation, activates factor XI to XIa. In the following step, factor XIa converts factor IX to IXa, which in turn activates factor X. This is the intrinsic coagulation pathway (Fig. 1). Conversion of factor X is the first common step in the coagulation cascade between the intrinsic and extrinsic pathways. The extrinsic pathway was traditionally felt to play a lesser role during extracorporeal circulation, due in part to the requirement for tissue injury and the subsequent exposure of tissue factor (TF), although TF may be present within extracorporeal circuits, in pro-inflammatory conditions, despite the absence of tissue injury [58]. The ability of activated complement to induce the expression of TF by monocytes may be one way in which this effect is mediated [59, 60]. Alternatively, Szotowski et al. have described the ability of TNF-α and IL-6 to induce the endothelial cell expression of soluble TF [61].

By whichever means the common pathway is arrived at, activated factor X (FXa) converts prothrombin to thrombin, which then cleaves fibrinogen to fibrin, resulting in subsequent clot formation. In addition to its role in coagulation, thrombin plays a major part in inflammation. It is known to increase the expression of P-selectin and E-selectin by endothelial cells, increasing neutrophil adherence and activation [62, 63]. Thrombin also induces endothelial cells to produce platelet activating factor (PAF), another potent activator of neutrophils [64], and directly influences neutrophils to express pro-inflammatory cytokines [65]. Attempts have been made to limit thrombin generation during extracorporeal circulation using biocompatible circuitry. Several coating materials are utilised in commercially available ECMO circuits, most commonly heparin and heparin-like compounds. Studies have demonstrated reduced levels of thrombin when heparin-bonded minimal extracorporeal circulation systems (MECC) are used, and these are functionally similar to ECMO [66, 67].

### Platelets

In addition to their role in haemostasis, platelets are a key mediator of inflammation during ECMO [68]. At the very outset of extracorporeal circulation, activated platelets begin to adhere to fibrinogen absorbed by the circuit. The activation of platelets largely occurs in response to thrombin generation, although complement activation [69] and the physical characteristics of the circuit also play a part. Adherent platelets undergo alterations in their shape and release their granular content (as do some circulating platelets). Platelet granules contain a variety of soluble mediators, including chemokines, pro-inflammatory cytokines, proteases, adhesion factors, growth factors, angiogenic factors and haemostatic factors [70, 71]. Bound platelets are also capable of forming leukocyte conjugates, predominantly with monocytes, but also neutrophils [72]. This platelet-leukocyte interaction induces leukocytes to secrete pro-inflammatory cytokines and monocytes to express TF [73, 74]. During ECMO, these processes appear to be time-dependent, with a progressive increase in activity noted in one study [68].

## ECMO and the complement system

The complement system acts at the forefront of the innate immune response and, as such, is one of the body’s first host defences to be activated when ECMO is initiated [23]. An interactive cascade of over 30 complement-related proteins acts to destroy and remove foreign substances identified as “non-self”, in addition to substances released from damaged cells. Complement achieves this either by direct cell lysis, by producing a trans-membrane pore known as a membrane attack complex (MAC), or by modulating leukocytes via opsonisation or the production of pro-inflammatory anaphylatoxins [75]. The complement cascade may be activated by any or all of three pathways: the classical pathway (CP), the alternative pathway (AP), and the lectin pathway (LP).

Across the three pathways, the first common step in the cascade is the cleavage of C3 to C3a and C3b (Fig. 1). In the alternative pathway, subsequent steps result in C5 being cleaved to form C5a and C5b. The two resulting molecules have distinct effects. C5a is a potent pro-inflammatory mediator (C3a is also an anaphylatoxin, albeit not as potent) with several abilities in vivo, including increasing leukocyte recruitment, increasing vascular permeability and inciting further inflammatory mediator release [76]. On the other hand, C5b progresses through several steps to produce C5b-9 (the MAC). Given its antibody independence, it is thought that the AP is the principal means of complement activation during extracorporeal circulation (ECC), occurring as a result of blood contacting the foreign material of the circuit [77].

Although the complement response to CPB has been well-described [78–81], the role of complement activation during ECMO is, by contrast, poorly defined. The few available studies date from the 1990s and were conducted using the much less advanced pump and circuit technology that was available to investigators at that time [19, 21–25]. This is important, as improvements in both circuitry and pump technology have likely had a beneficial effect on the levels of complement activation seen during ECMO. Studies on CPB have shown us that the use of centrifugal pumps, now ubiquitous in modern ECMO, reduces complement activation [82, 83]. In addition, the use of contemporary heparin-bonded circuits has also been shown to reduce complement activation [84]. Studies have examined complement activation in ex-vivo models of ECMO [20, 22, 24], neonatal ECMO [20, 23, 25] and in adult patients [21]. In keeping with CPB, all of these studies revealed rapid elevations in the levels of complement factors during ECMO, with peaks occurring within 1 to 2 hours.

As previously stated, a key differentiator between the extracorporeal circulation of CPB and ECMO is their relative duration. This effect has been described by several authors. In two adult patients on ECMO for acute respiratory distress syndrome (ARDS), Vallhonrat et al., described rapid elevations in the levels of C3b and terminal complement complex (TCC) (fluid-phase MAC), which were evident within 15 minutes of commencing ECMO. These levels peaked by 60 minutes and had begun to fall by 180 minutes, reaching near-baseline levels at 2 days. Of note was the failure to demonstrate any significant elevation in C4d, a classical pathway marker [21]. In neonates, Graulich et al. detected peaks of C3a at 1 hour and of C5a and TCC at 2 hours. However, contrary to what Vallhonrat et al. observed, TCC levels did not return to baseline [20].

Ex-vivo studies of complement activation in ECMO have almost universally failed to uncover peak or falling levels of complement products. Using heparinised fresh whole human blood in an ex-vivo circuit, Moen et al. recorded levels of C3 activation products and TCC continuing to rise after 72 hours of ECMO [22]; this points towards the presence of a negative feedback mechanism in-vivo, which acts to limit inappropriate complement activation. Bergman et al. employed an ex-vivo model of ECMO, with a mixture of non-heparinised citrated fresh human blood and Ringer’s solution, to examine the influence of complement activation during ECMO on blood cell rheological properties. Close correlation between the levels of complement factors, particularly C5a and TCC, and white cell deformability were noted, leading the authors to conclude that high levels of complement activation during ECMO may play a part in the rheological deterioration of blood cells [24].

## ECMO, the endothelium and leukocytes

### Endothelial cell activation

Endothelial dysfunction in the critically ill is an important marker of poor outcome [85, 86]. Even without being in direct contact with the extracorporeal circuit, the vascular endothelium plays a crucial role in the inflammatory response during ECMO. In a fashion similar to that of SIRS, the generation of inflammatory mediators in response to extracorporeal circulation results in the widespread activation of the endothelium, with a subsequent alteration in endothelial cell gene expression [87]. This occurs in response to a number of cytokines, complement products and reactive oxygen species (ROS). Activated endothelial cells in turn secrete pro-inflammatory cytokines and increase their expression of adhesion molecules, leading to the increased trans-migration of leukocytes.

Early activation of the endothelium occurs in response to complement products [88]. These factors act on endothelial cells to induce the upregulation of P-selectin. The expression of P-selectin on the endothelial surface in turn spurs the recruitment of activated leukocytes. This process occurs rapidly, as P-selectin exists pre-stored in the cytoplasm of endothelial cells. However, complement-mediated endothelial activation is short-lived, thus, it is an increase in the circulating levels of pro-inflammatory cytokines that is responsible for the later activation of the endothelium [87]. TNF-α and IL-1β are perhaps the most influential cytokines in this process [36]. Endothelial activation by either mechanism results in the recruitment, “rolling”, firm adhesion and trans-migration of activated neutrophils. Neutrophilic infiltration is thought to be responsible for the end-organ damage associated with ECMO [15].

### Neutrophils and monocytes

Neutrophils are activated by several mechanisms triggered by ECMO. This response shares certain similarities with the pathogenesis of ARDS [89]. Complement contributes to the almost instantaneous activation of neutrophils on the commencement of extracorporeal circulation [90]. Using a simulated model of ECC, Rinder et al. have described the importance of C5a and TCC in this process, showing that the addition of a human monoclonal antibody directed at C5 significantly inhibits neutrophil activation [91].

Ex-vivo models of ECMO show that the activation of neutrophils increases dramatically within the first 30 minutes, peaks within the first few hours, and declines thereafter. Of note from these studies, is the observation that leukocytes do not adhere to the circuitry [26, 27]. Once activated, neutrophils degranulate, releasing stores of cytotoxic enzymes, such as neutrophil elastase, myeloperoxidases and lysozymes. In addition, via a “respiratory burst”, neutrophils are also capable of releasing cytotoxic ROS. It is via these mechanisms, both within the vasculature and after trans-migration into the tissues that neutrophils act as the effectors of organ damage during ECMO [92]. The site of neutrophil activation during ECMO is likely to be multifocal, although in one experimental model of ECMO the fundamental role of the oxygenator was described [14]. During CPB, concentrations of neutrophil elastase, IL-8 and neutrophils are significantly elevated in bronchoalveolar lavage fluid; furthermore, these levels are correlated with the patients’ arterial partial pressure of oxygen (PaO_2_)/fraction of inspired oxygen (FiO_2_) ratios [93].

While no comparable study has been conducted in adults on ECMO, early studies, performed in neonates on ECMO, demonstrated an association between neutrophil activation and pulmonary deterioration [23, 27]. Increased neutrophil margination into several tissues, particularly the lungs, has been described during ECMO, both in animal models and in neonates [14, 15, 30, 94]. Overall, neutrophil numbers increase after the initiation of extracorporeal circulation [95] and neutrophil function appears to be preserved [25, 28]. This is despite the reported adverse effects of ECMO on leukocyte rheology [96].

Monocytes are also activated in response to ECMO [26], though this appears to occur more slowly than the activation of neutrophils [36]. When appropriately stimulated, monocytes may secrete a number of pro-inflammatory cytokines [97].

## ECMO and cytokines

The initiation of ECMO results in the production of a variety of pro-inflammatory and anti-inflammatory cytokines. These small proteins have a variety of roles in cell signalling and are important mediators of the innate immune response. There is little evidence to support a single “master” cytokine in this process. This has implications in selecting potential therapeutic interventions. While interventions aimed at single cytokine targets are unlikely to overcome the redundancy in the system, more broad-based approaches, such as cell therapy, appear to have significant potential. We subsequently describe the most studied cytokines with respect to ECMO.

TNF-α is produced by a range of cell types in response to an inflammatory stimulus. TNF-α has pleiotropic effects on a variety of tissues; but, perhaps most importantly, it is a potent activator of neutrophils [98]. Furthermore, it induces the expression of adhesion molecules by endothelial cells, enhancing the margination of neutrophils. TNF-α can also stimulate macrophage phagocytosis, enhance the expression of prostaglandins and increase the formation of thrombin [99]. TNF-α acts as a negative inotrope at the macrocirculatory level. During ECMO, it is thought that a large proportion of early TNF-α release originates from pre-formed stores in mast cells. In a porcine ECMO study, McIlwain et al. identified gut mucosal mast cells as the key contributor to early TNF-α release. During the first 2 hours of ECMO, increased plasma TNF-α was not associated with increased tissue synthesis. This, combined with a decreased level of TNF-α found in gut mucosa, led the authors to hypothesise that mast cell degranulation, in response to complement activation, is the likely mechanism by which TNF-α concentrations are elevated [15]. Higher levels of TNF-α have been associated with non-survival in neonates undergoing ECMO [23, 27].

Interleukin-6 is a complicated cytokine, with both pro- and anti-inflammatory actions. Whilst promoting the acute phase reaction, expansion and activation of T-cells and the differentiation of B cells, IL-6 may also down-regulate the expression of other pro-inflammatory cytokines, as well as up-regulate the expression of the anti-inflammatory cytokines [100]. Studies have reported consistent elevations in IL-6 levels during ECMO [15, 18, 30, 32, 101]. IL-6 levels in the lungs have also been shown to rise after VV-ECMO in an animal model, where they were associated with parenchymal damage [30]. Interestingly, given that IL-6 has a key role initiating the acute phase response and its concentration is thus usually closely coupled to C-reactive protein (CRP) production, McIlwain et al. failed to demonstrate any link between the two during porcine ECMO [15]. In a combined study by Risnes et al., involving children and adults supported with ECMO, levels of IL-6 were inversely associated with survival, with a clear divergence in IL-6 concentrations between survivors and non-survivors after two days of ECMO support: survivors effectively normalised their IL-6 levels whilst those who died maintained a persistent elevation. This was not observed to be the case for other cytokines, such as IL1-β, IL-8 or IL-10 [102].

IL-8 is a potent neutrophil activator and a chemoattractant for neutrophils, basophils and T-lymphocytes. Studies using simulations and animal models and in neonates have demonstrated rapid elevations of IL-8 after ECMO commences [15, 18, 27]. Alterations in IL-8 concentration appear to follow a temporal trend similar to TNF-α, with increases noted within the first 15 minutes of ECMO [15, 27].

A number of other cytokines have also been implicated in the inflammatory response to ECMO and extracorporeal circulation. Of note is that low levels of the anti-inflammatory cytokine IL-10 (at the time of ECMO initiation) have been associated with poor survival [103].

## Future directions

In the last decade, ECMO technology has improved dramatically. This has facilitated its uptake in an increasing number of centres for an ever-growing range of indications. Ongoing research into anticoagulation, servo control and membrane lung construction will further improve the safety and efficacy of ECMO [104]. Additional research (pre-clinical and clinical) is required to assess the impact of ECMO-induced inflammation on clinically meaningful outcomes. This work will inform the requirement for novel anti-inflammatory therapies that could be used during ECMO. In future, when designing strategies to minimise the inflammatory response to ECMO, several experimental treatments appear to have potential, in particular, the use of Factor XII inhibitors and mesenchymal stromal cell (MSC) therapy.

Currently, there is substantial interest in targeting factor XII as a novel means of anticoagulation [105]. The use of a factor XII inhibitor in this setting is attractive given the potential to reduce, or eliminate, the risk of thrombosis without encountering the bleeding risk associated with current anticoagulants. A beneficial side-effect would be the inhibition of contact-mediated inflammation involving the activation of factor XII. A recombinant human antibody, 3 F7, which inhibits FXIIa, has been successfully tested in a rabbit model of VA-ECMO [55]. Any future work will need to demonstrate the safety and efficacy of factor XII inhibitors in a large animal model of ECMO, using contemporary clinical ECMO equipment, before progression to human studies.

MSC therapy is being investigated as a treatment for a number of acute inflammatory conditions. MSCs are multipotent adult stem cells found in most tissues of mesodermal origin, such as bone marrow [106]. MSCs have attracted attention due to their immunomodulatory effects. In-vitro MSCs have been shown to influence both the adaptive and innate immune response to infection and inflammation; this ability is bi-directional, that is to say, MSCs may either promote or ameliorate inflammation dependent on the contemporary milieu [107]. MSCs have been studied in a pre-clinical model of CPB as a means of reducing the harm associated with ischaemia-reperfusion injury [108]. Here, infusion of human MSCs significantly reduced the levels of inflammatory cytokines within 3 hours. Given their ability to influence the innate immune response at a range of points in the cascade and in a manner consistent with the local environment, MSCs have the potential to be efficacious in the setting of ECMO-induced inflammation.

## Conclusion

The inflammatory response to ECMO is complex and multi-faceted. It remains unclear whether this excess inflammation is all deleterious, or if it has potential benefits to the host. In summary, it arises principally due to the contact and complement systems becoming activated as a result of blood exposure to the extracorporeal circuit. The combination of a sustained innate immune response and the pro-inflammatory aspects of coagulation result in “pan-endothelial” injury, with leukocyte activation and the production of pro-inflammatory mediators. This ultimately ends in a systemic inflammatory response and end-organ damage.

Whilst this process shares a number of similarities with the inflammatory response witnessed during CPB, there are also a number of important distinctions. Given the recent increase in the use of ECMO, there is a relative paucity of data on the inflammatory response that it induces. This is compounded by the fact that many of the available studies were conducted prior to the introduction of modern innovations in ECMO technology. Mechanistic and outcome information from adult patients is absent. Clearly all patients supported on ECMO experience some degree of inflammatory response to ECMO, but currently there is no physiological rationale to explain the extreme variation seen in the magnitude of the patients’ responses to that inflammation, nor their clinical course. Whilst it is undoubtedly difficult to isolate the relative contribution of specific components of the inflammatory response (pro-inflammatory and CARS) to outcomes after ECMO, failure to do so may lead to a missed opportunity for intervention. The first step towards addressing this deficit is to not only improve our understanding of the basic science underpinning the inflammatory response induced by modern ECMO, but to adopt minimal reporting criteria for the inflammatory response during human ECMO studies, in a manner similar to that proposed for CPB [109]. Such studies should be conducted in models that are relevant, using technology that is in contemporary clinical use. A more detailed understanding of the underlying processes will allow us to better investigate potential therapies and design future clinical studies.

## Abbreviations

AP: Alternative (complement) pathway
ARDS: Acute respiratory distress syndrome
CABG: Coronary artery bypass grafting
CARS: Compensatory anti-inflammatory response syndrome
CP: Classical (complement) pathway
CPB: Cardiopulmonary bypass
ECC: Extracorporeal circulation
ECMO: Extracorporeal membrane oxygenation
ECPR: Extracorporeal cardiopulmonary resuscitation
ELSO: Extracorporeal Life Support Organisation
FiO_2_: Fraction of inspired oxygen
HMWK: High molecular weight kininogen
IL: Interleukin
LP: Lectin (complement) pathway
MAC: Membrane attack complex
PAF: Platelet activating factor
PaO_2_: Arterial partial pressure of oxygen
ROS: Reactive oxygen species
SIRS: Systemic inflammatory response syndrome
TCC: Terminal complement complex
TF: Tissue factor
TNF-α: Tumour necrosis factor alpha
VA: Veno-arterial
VV: Veno-venous
